# Correction to: High-resolution ISR amplicon sequencing reveals personalized oral microbiome

**DOI:** 10.1186/s40168-018-0594-1

**Published:** 2018-11-20

**Authors:** Chiranjit Mukherjee, Clifford J. Beall, Ann L. Griffen, Eugene J. Leys

**Affiliations:** College of Dentistry, The Ohio State University, Columbus, OH USA

## Correction

Following publication of the original article, the authors recognized that the left and right panels in Fig. 6b had been inadvertently switched during reformatting. As a result, while the axis labels referred to the correct organism, they did not correspond to the headings. The corrected figure is given below:

**Fig. 6:**
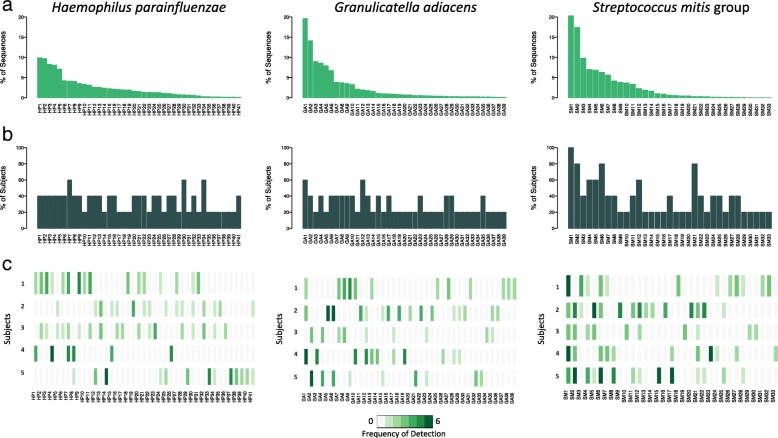
Population structure for three abundant oral bacteria. **a** For each species, bar plot of relative abundance for the entire dataset is shown. The ISR types are ranked and labelled in order of decreasing abundance, and the same order is used in the lower panels. **b** Prevalence among subjects, determined by presence at any time point, is shown as bar graphs. **c** Frequency of detection of ISR types over time within subjects is shown using a heat map

