# Supplementary material for: High-resolution ISR amplicon sequencing reveals personalized oral microbiome
Source: Microbiome. 2018 Sep 5;6:153. doi: 10.1186/s40168-018-0535-z (PMC6126016; doi:10.1186/s40168-018-0535-z)
Supplement: Supplementary file 2 — Table S1. Primers used in this study. (DOCX 25 kb) [file 40168_2018_535_MOESM2_ESM.docx]

**Table S1 Primers used in this study.**

| **Locus** | **Name** | **Primer Sequence** | **Target** | **Reference** |
| --- | --- | --- | --- | --- |
| V1-V3 | 27f-YM* | AGAGTTTGATYMTGGCTCAG | General V1 forward | [1] |
|  | 27f-Chl* | AGAATTTGATCTTGGTTCAG | *Chlamydiales* |  |
|  | 27f-Bor* | AGAGTTTGATCCTGGCTTAG | *Borrellia* |  |
|  | 27f-Bif* | AGGGTTCGATTCTGGCTCAG | *Bifidobacteriaceae* |  |
|  | 534r | ATTACCGCGGCTGCTGG | General V3 reverse | [2] |
|  |  |  |  |  |
| V8- | 1237f** | GGGCTACACACGYGCWAC | General V8 forward | [3] |
| 23S | EricM** | GCCWAGGCATCCDCC | General 23S reverse | [4] |

*These were mixed in 3:1:1:1 ratio. **Modified from original for this study.

Supplementary References:

1. Frank JA, Reich CI, Sharma S, Weisbaum JS, Wilson BA, Olsen GJ. Critical evaluation of two primers commonly used for amplification of bacterial 16S rRNA genes. Appl Environ Microbiol. 2008;74:2461–70.

2. Muyzer G, De Waal EC, Uitterlinden AG. Profiling of complex microbial populations by denaturing gradient gel electrophoresis analysis of polymerase chain reaction-amplified genes coding for 16S rRNA. Appl Environ Microbiol. 1993;59:695–700. doi:0099-2240/93/030695-06$02.00/0.

3. Turner S, Pryer KM, Miao VPW, Palmer JD. Investigating deep phylogenetic relationships among cyanobacteria and plastids by small subunit rRNA sequence analysis. J Eukaryot Microbiol. 1999;46:327–38.

4. Rumpf RW, Griffen AL, Wen BG, Leys EJ. Sequencing of the ribosomal intergenic spacer region for strain identification of Porphyromonas gingivalis. J Clin Microbiol. 1999;37:2723–5.
