# Supplementary figures and images for: High-resolution ISR amplicon sequencing reveals personalized oral microbiome
Source: Microbiome. 2018 Sep 5;6:153. doi: 10.1186/s40168-018-0535-z (PMC6126016; doi:10.1186/s40168-018-0535-z)

## ISR Blast Matches

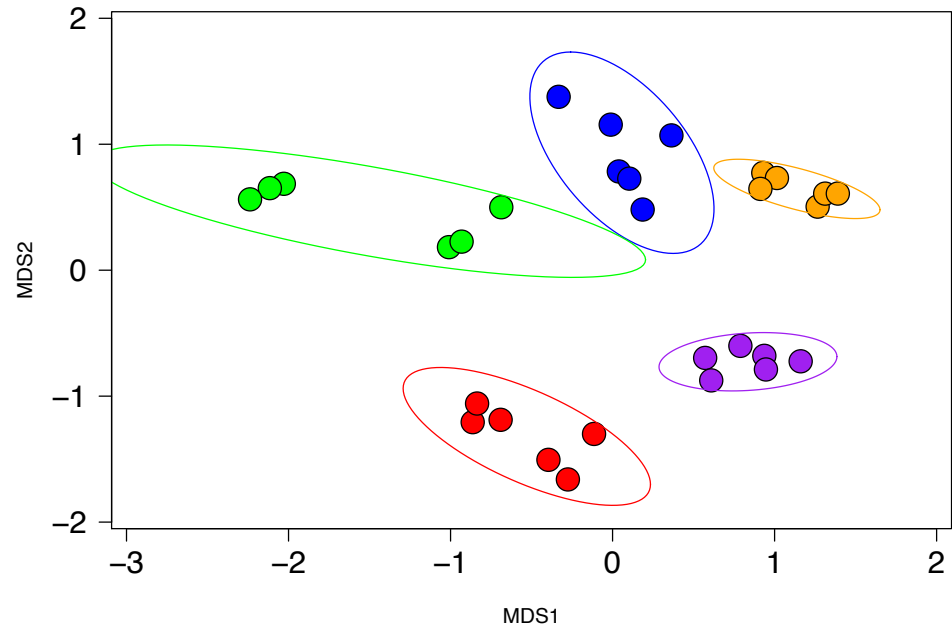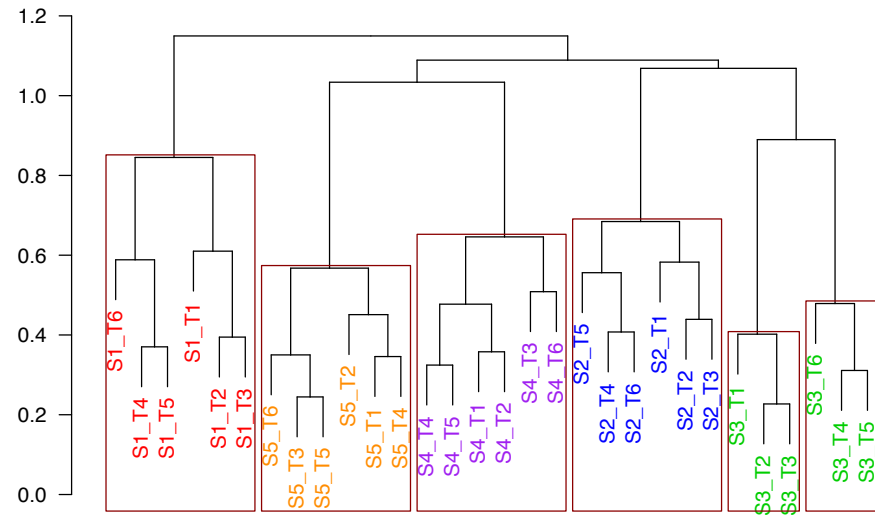

Supplement: Supplementary file 4 — Figure S1. Distance-based community analysis. (PDF 58 kb) [file 40168_2018_535_MOESM4_ESM.pdf]

*Haemophilus parainfluenzae*

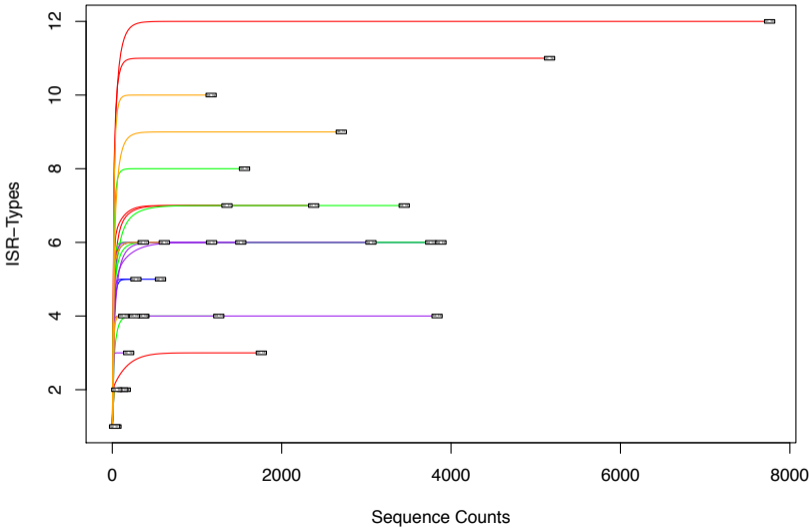

*Granulicatella adiacens*

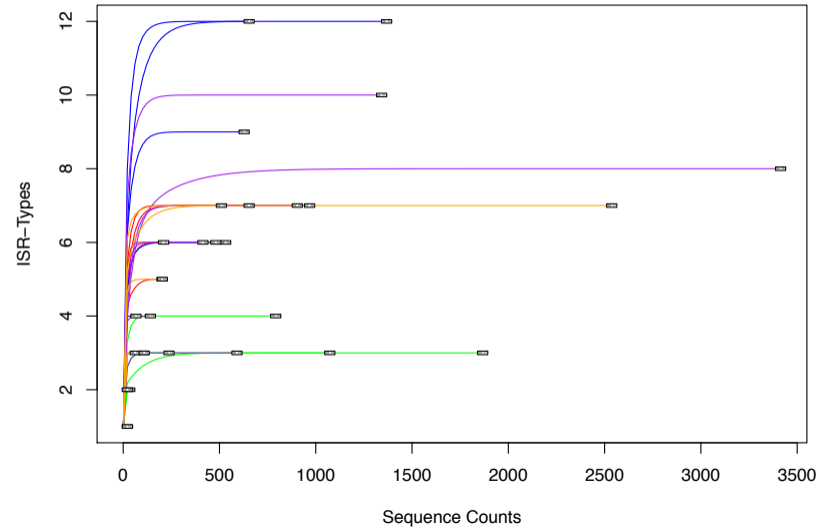

*Streptococcus mitis* group

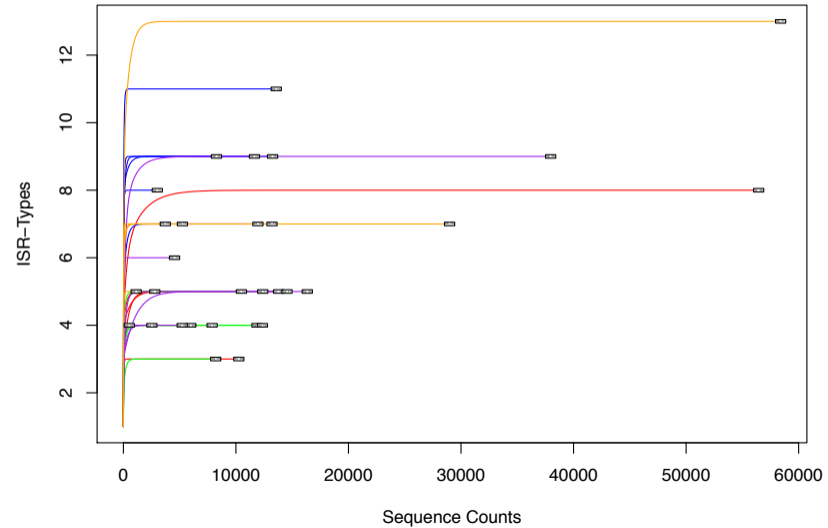

Supplement: Supplementary file 5 — Figure S2. Rarefaction curve for the three most diverse species. (PDF 186 kb) [file 40168_2018_535_MOESM5_ESM.pdf]
